# Addition of Riboflavin-Coupled Magnetic Beads Increases Current Production in Bioelectrochemical Systems via the Increased Formation of Anode-Biofilms

**DOI:** 10.3389/fmicb.2019.00126

**Published:** 2019-02-05

**Authors:** Tutut Arinda, Laura-Alina Philipp, David Rehnlund, Miriam Edel, Jonas Chodorski, Markus Stöckl, Dirk Holtmann, Roland Ulber, Johannes Gescher, Katrin Sturm-Richter

**Affiliations:** ^1^Institute for Applied Biosciences, Department of Applied Biology, Karlsruhe Institute of Technology, Karlsruhe, Germany; ^2^Chair of Bioprocess Engineering, Technical University of Kaiserslautern, Kaiserslautern, Germany; ^3^Electrochemistry, DECHEMA-Forschungsinstitut, Frankfurt, Germany; ^4^Industrial Biotechnology, DECHEMA-Forschungsinstitut, Frankfurt, Germany; ^5^Institute for Biological Interfaces, Karlsruhe Institute of Technology, Karlsruhe, Germany

**Keywords:** *Shewanella oneidensis*, bioelectrochemical systems, electron shuttle, flavins, magnetic beads, extracellular electron transfer

## Abstract

*Shewanella oneidensis* is one of the best-understood model organisms for extracellular electron transfer. Endogenously produced and exported flavin molecules seem to play an important role in this process and mediate the connection between respiratory enzymes on the cell surface and the insoluble substrate by acting as electron shuttle and cytochrome-bound cofactor. Consequently, the addition of riboflavin to a bioelectrochemical system (BES) containing *S. oneidensis* cells as biocatalyst leads to a strong current increase. Still, an external application of riboflavin to increase current production in continuously operating BESs does not seem to be applicable due to the constant washout of the soluble flavin compound. In this study, we developed a recyclable electron shuttle to overcome the limitation of mediator addition to BES. Riboflavin was coupled to magnetic beads that can easily be recycled from the medium. The effect on current production and cell distribution in a BES as well as the recovery rate and the stability of the beads was investigated. The addition of synthesized beads leads to a more than twofold higher current production, which was likely caused by increased biofilm production. Moreover, 90% of the flavin-coupled beads could be recovered from the BESs using a magnetic separator.

## Introduction

In microbial fuel cells, microorganisms catalyze the direct conversion of chemical energy into an electrical current. This ability can be used in a variety of biotechnological processes like wastewater treatment or the production of platform chemicals. The biocatalytic microorganisms of this process are characterized by the ability to transfer respiratory electrons to the cell surface and subsequently onto solid state electron acceptors like for instance ferric iron minerals or, as a synthetic variant, an anode surface (Nealson et al., 2002; Logan, 2009; Javed et al., 2018).

*Shewanella oneidensis* (MR-1) and *Geobacter sulfurreducens* (PCA) are the two main model microbes for exploring molecular mechanisms of extracellular electron transfer (EET) (Nealson and Rowe, 2016; Simonte et al., 2017; Beblawy et al., 2018). The pathway of electron flow toward the extracellular space is similar in MR-1 and PCA (Shi et al., 2014). Both organisms are Gram-negative, which means that respiratory electrons are transported from the cytoplasmic membrane through the periplasm and across the outer membrane in order to be transferred onto insoluble electron acceptors like metals, iron minerals or an anode (Simonte et al., 2017; Beblawy et al., 2018; Costa et al., 2018). *C*-type cytochromes with multiple heme centers are the players in this process and electron transfer through the outer membrane is conducted via a three-protein complex consisting of one cytochrome on the periplasmic side and a second, surface-oriented cytochrome on the cell surface. An integral β-barrel protein connects the two cytochromes which leads to a *trans*-outer membrane porin-cytochrome complex (White et al., 2016; Chong et al., 2018; Costa et al., 2018). Several mechanisms of terminal electron transfer from the outer membrane cytochromes (OMCs) to the electron acceptor were proposed. This includes either direct contact of enzymes and acceptor (White et al., 2016; Simonte et al., 2017), reduction via conductive extracellular appendages (so called nanowires) (Reguera et al., 2005; Gorby et al., 2006; Malvankar et al., 2011; Polizzi et al., 2012) or mediated transfer through the reduction of electron carriers [usually small, low-weight, soluble redox molecules (e.g., phenazine and quinones)] in a process termed electron shuttling (Lovley et al., 1998; Okamoto et al., 2015).

The precise role of shuttles in EET is still under debate. Currently, researchers operate with two hypotheses. The first hypothesis acts on the assumption that indirect EET via shuttling compounds is a major factor in MR-1 and electron transfer in PCA exclusively depends on direct interaction with the electron acceptor. Evidence for this is especially based on results from BES experiments with supernatant exchange. Here, after the replacement of spent with fresh medium, MR-1 exhibits a prompt current drop and only starts to produce current after a lag phase – whereas an exchange of the supernatant of PCA cultures impacts current production only negligible (Bond and Lovley, 2003; Marsili et al., 2008; Okamoto et al., 2014). Furthermore, Gralnick and colleagues demonstrated that in MR-1 EET is based on self-secreted redox-active compounds to around 80% and the electron carriers were determined to be flavins, namely riboflavin (vitamin B_2_) and flavin mononucleotide (FMN) (Marsili et al., 2008; Brutinel and Gralnick, 2012; Kotloski and Gralnick, 2013). However, compared to other redox mediators like humic acids or phenazines, flavins enhance EET much faster and at lower concentrations (Marsili et al., 2008). Presumably, the role of flavins can be expanded from an electron shuttle to a cytochrome-bound cofactor that enhances the kinetics of electron transfer much faster than as a shuttling compound. Hence, the second hypothesis on the role of flavins in EET relies on a binding of flavins to the OMCs as cofactors and by this suggests a similar mechanism for both organisms. The species-dependent binding affinities of OMCs from MR-1 and PCA to flavin provide an explanation for the electrochemical differences between MR-1 and PCA in the above-described medium exchange experiment, as they seem to bind much stronger to PCA OMCs compared to the ones of MR-1 (Xu et al., 2016; Babanova et al., 2017).

In experiments based on the reduction of insoluble electron acceptors like metals, redox shuttles are often added in micromolar concentrations. However, natural secretion of flavins by MR-1 is much lower and seems to be dependent on various factors like growth phase, medium, electron acceptors, the BES setup and other experimental conditions. Already in Marsili et al. (2008) showed that riboflavin accumulates in the supernatant of 4-day-old biofilms in concentrations of 250 to 500 nM (Marsili et al., 2008). Other groups measured only 25 nM flavin in the bulk solution and a change of the BES setup resulted in a different concentration of riboflavin (Velasquez-Orta et al., 2010; Zhai et al., 2016; Lu et al., 2017). Independent of the natural concentration, the artificial addition of riboflavin has stimulatory effects in a BES and this seems to be linearly dependent on the riboflavin concentration in a certain concentration range. Consequently, several groups tried to raise the riboflavin concentration either via genetic engineering of MR-1 itself or via co-culturing with other riboflavin producing strains (Zhai et al., 2016; Lu et al., 2017). Still, from an application point of view, although bioelectrochemical systems (BESs) are perfectly suited for continuous biofilm based biotechnological production, the constant stream of fresh media over the biofilm would result in a continuous wash out of flavin compounds. Hence, neither the exogenous addition of soluble flavins nor the endogenous overproduction seem to be key strategies to enhance biotechnological production or consumption rates.

The aim of this research was to study the application of a recyclable electron shuttle and its effect on current production in a BES in order to overcome disadvantages and limitations of flavin addition such as losses by washing out or high process costs. Therefore, riboflavin was coupled to magnetic beads and the effects on current production in MR-1 as well as their stability and recyclability were investigated.

## Materials and Methods

### Strains and Media

All strains used in this study are listed in Table 1. *S. oneidensis* barcode is a strain that was developed by Dolch et al. (2016). It contains a 72 bp synthetic genomic integration that can be used for the quantification of cells via quantitative PCR (qPCR, see below). The *gfp* strain constitutively expresses a *gfp* gene and was used for fluorescence microcopy (Stöckl et al., 2016). All strains were pre-cultured under oxic conditions in LB medium at 30°C. Cells were then transferred into anoxic minimal medium containing 12 mM HEPES buffer, 70 mM lactate as electron donor and 100 mM fumarate as electron acceptor. Furthermore, the medium contained (per liter): 0.221 g K_2_HPO_4_, 0.099 g KH_2_PO_4_, 0.168 g NAHCO_3_, 1.189 g (NH_4_)_2_SO_4_, and 8.77 g NaCl supplemented with 1 mM MgSO_4_, 0.1 mM CaCl_2_, 1 g casamino acids, and trace elements (5 μM CoCl_2_, 0.2 μM CuSO_4_, 57 μM H_3_BO_3_, 5.4 μM FeCl_2_, 1.3 μM MnSO_4_, 67.2 μM Na_2_EDTA, 3.9 μM Na_2_MoO_4_, 1.5 μM Na_2_SeO_4_, 5 μM NiCl_2_, and 1 μM ZnSO_4_). The pH was adjusted to 7.4 and all bottles were sealed with rubber stoppers. Prior to autoclaving, oxygen was removed from the medium via the repeated application of a vacuum and following sparging of the headspace with N_2_. Before inoculation into bioelectrochemical reactors, the cells were harvested by centrifugation (5 min, 6000 g) and washed twice with medium containing neither electron donor nor electron acceptor. Thereafter, the cells were resuspended to an OD_600_ of 0.07 in medium containing 70 mM lactate but no electron acceptor. The *gfp*-strain was cultivated with the addition of kanamycin (50 mg L^-1^) and L-rhamnose (2.2 g L^-1^) for plasmid maintenance and continuous eGFP expression, respectively.

**Table 1:** Strains used in this study.

Strain	Source or reference
*Shewanella oneidensis* MR-1 wild type	DSMZ
*Shewanella oneidensis* MR-1 barcode	Dolch et al., 2016
*Shewanella oneidensis* MR-1 *gfp*	Stöckl et al., 2016
*Shewanella oneidensis* MR-1 ΔOMC	Bucking et al., 2012


### BES Experiments

All bioelectrochemical experiments were conducted in triplicates using a single chamber MFC with a working volume of 270 ml (Bursac et al., 2017). Graphite felt (projected area of 36 cm^2^, SGL Group, Germany) and platinum mesh (projected area of 1.25 cm^2^, chemPUR, Germany) were used as anode and cathode material, respectively, and an Ag/AgCl electrode (Sensortechnik Meinsberg, Germany) was used as reference electrode. Before use, the anode was first rinsed with isopropanol, followed by deionized water. The complete bioelectrochemical setup was sterilized by autoclaving. During chronoamperometric experiments, the working electrode was poised to 0 mV vs. SHE using a potentiostat (Pine Instruments, United States) and current was monitored for 46 h. BES were constantly flushed with N_2_ gas in order to provide anoxic conditions and constant mixing of the liquid phase.

### Magnetic Beads Preparation and Determination

The magnetic beads (PureCube MagBeads; Cube Biotech, Germany) used in this work are spherical magnetic agarose beads, consisting of 6% cross-linked agarose. The beads were synthesized by the company as a solid phase-based material via EDC/NHS-mediated coupling for covalent modification. A riboflavin-modified epoxide function was coupled to the magnetic agarose with three different alkane carbon chains with a respective chain length of 4 (C4), 11 (C11), and 40 (C40) carbon atoms. An alternative, glycine-modified epoxide function was used as a control and is from here on referred to as *glycine-coupled beads*. The beads had an average particle diameter of 25 μm and were stored in a 25% (w/v) suspension in 100% isopropanol. Prior to usage, the beads were rinsed three times with sterile medium without electron donor and acceptor as stated above.

The number of beads was quantified using a Neubauer chamber (Roth, Germany). The recovering process from the anolyte of the BES was performed using a magnetic separator and the recovery efficiency was calculated based on the difference between particle number added before the bioelectrochemical experiments and after the recovering process at the end of the experiments.

### Riboflavin Quantification

Riboflavin was decoupled from the beads chemically by adding 100 mM NaOH for 30 min. The beads were then separated from the riboflavin solution using a magnetic separator. Riboflavin was quantified using a plate reader (Infinite Pro M200 Tecan, Switzerland) at 440 nm (Bartzatt and Wol, 2014). The riboflavin concentration was determined based on a calibration curve. Several dilutions of a riboflavin solution in 100 mM NaOH with concentrations ranging from 0.5 to 10 μM were prepared as standards.

### DNA Isolation and Quantitative PCR (qPCR)

Genomic DNA isolation as well qPCR based cell quantification were conducted according to Dolch et al. (2016).

### Binding of Riboflavin to OMCs

Wild type and ΔOMC cells were pre-grown, harvested and centrifuged for 5 min at 6000 g. The optical density was adjusted in minimal medium with 50 mM lactate as electron donor and 10 mM fumarate as electron acceptor to OD_600_ of 1. 1 ml of cells was mixed with 100 μl of beads (coupled to either riboflavin or glycine) and cells were incubated overnight under anoxic conditions. Subsequently, the beads were washed twice in anoxic medium lacking electron donor and acceptor using a magnetic separator. The binding of cells was assessed qualitatively under the microscope.

### Statistical Analysis

Significance of the data was determined via Welch Two Sample *T*-test and *F*-test. All data sets were normally distributed. The level of significance was set to 5%.

### Flow Cell Experiments and Electrochemical Impedance Spectroscopy (EIS)

Experiments in the impedance flow cell were performed according to Stöckl et al. (2016) in a previously described flow cell (40). The flow cell was modified by the addition of an Ag/AgCl reference electrode (RE-3VT, ALS, Tokyo, Japan) and assembled under sterile conditions. Sterile Na_2_SO_4_ [electrolyte counter electrode (CE)] and lactate containing medium [LM, for working electrode (WE)] were filled into the respective half-cell chambers. The flow cells were tempered under an incubation hood to 30°C and the solutions were pumped through the flow cell by a peristaltic pump. The WE solution was flushed with N_2_ with a flow rate of 5 mL min^-1^ in order to ensure anoxic conditions in the WE chamber. The polarization and EIS routines were subsequently started.

A potential (E) of -199 mV vs. Ag/AgCl (0 mV vs. SHE) was applied to the indium tin oxide (ITO) WE and EIS was measured with a potentiostat (Reference 600, Gamry Instruments, United States).

The frequency (f) range varied between 100 kHz and 50 mHz with an amplitude (U) of 10 mV rms and 10 points per decade. Polarization of the WE was started prior to the addition of bacteria to an OD_600_ of 0.1. Beads were added simultaneously to the cells to the WE chamber. Similar to the bacteria the beads were circulated between the flow cell and WE chamber reservoir. EIS measurements were performed after 24 h of incubation.

### Fluorescence Microscopy

Microscopic images of cells growing on the ITO WE were taken 24 h after inoculation. Samples were viewed on a Leica DM 5500B and images were taken with a Leica DFC 360 FX camera and the corresponding Leica LAS AF Lite software. Pumping was paused during image acquisition to prevent image disturbance by the peristaltic pump.

## Results

### Effect of Riboflavin Coupled Magnetic Beads on Current Production

**FIGURE 1:**
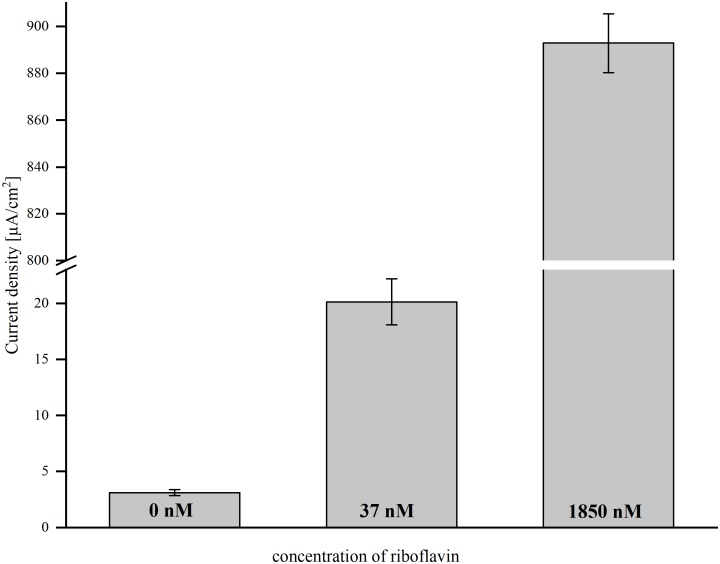
The effect of direct riboflavin addition on average current density in BES with 0, 37, and 1850 nM riboflavin. Error bars represent the standard deviations from means of samples taken in independent triplicates.

The aim of this study was to evaluate the possibility to increase current densities in BESs using riboflavin-coupled magnetic beads. To find a suitable working concentration, the effect of a low riboflavin concentration (as a free, soluble compound) on current production in a BES inoculated with MR-1 was tested first. Current was monitored over time and the average current density was compared after 46 h. Without the addition of riboflavin, the cells produced an average current density of 3.1 (±0.28) μA/cm^2^. The addition of 37 nM riboflavin enhanced current density 6.5-fold and resulted in 20.1 (±2.05) μA/cm^2^ (Figure 1). A 50-fold higher shuttle concentration resulted in a linear increase of the current density, but for economic reasons we chose 37 nM riboflavin as suitable working concentration for further experiments.

**FIGURE 2:**
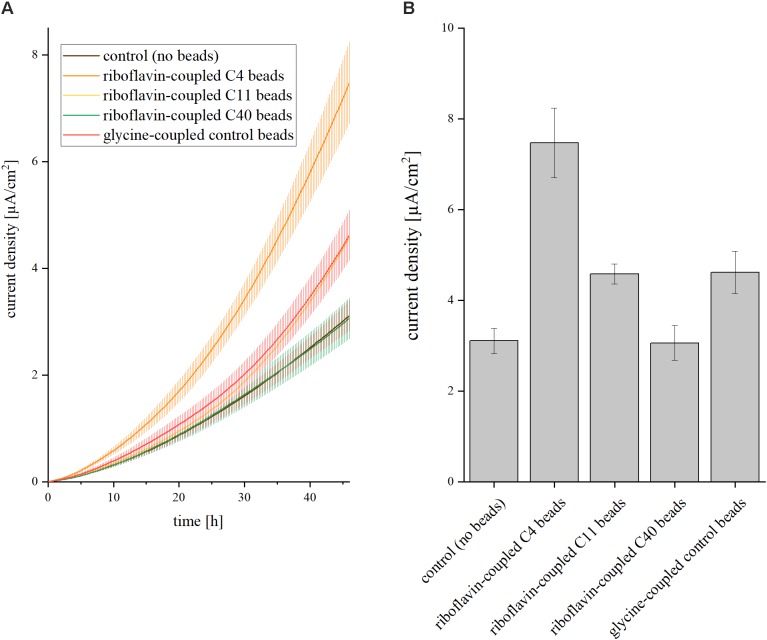
Impact of riboflavin coupled beads on current density in BES. Three different linker molecules characterized by short (C4), medium (C11), and long (C40) chain length were placed between beads and the riboflavin molecule. The control experiment was conducted without addition of riboflavin or magnetic beads; C4/C11/C40 represent the addition of riboflavin-coupled beads with the respective linker chain lengths; glycine instead of a riboflavin molecule was added to the C4 linker in the glycine-coupled control beads. The concentration of riboflavin was 37 nM in all experiments. **(A)** Current density over 46 h. Error bars represent the standard deviations from means of samples taken in independent triplicates. **(B)** Average current density after 46 h of operation. Error bars represent the standard deviations from means of samples taken in independent triplicates.

The next step was to transfer the effect of soluble riboflavin on the current density to a recyclable shuttle that can be extracted easily from the medium. Therefore, riboflavin was coupled to magnetic beads via organic spacers of different chain lengths: short (C4), medium (C11), and long (C40). The number of C-atoms in the spacer had a strong impact on the current density in the BES (Figure 2). Short spacers resulted in the highest current densities [7.5 ( ± 0.76) μA/cm^2^]. With increasing number of C-atoms between riboflavin and the magnetic beads, the current production dropped. Medium spacers exhibited a current of 4.6 (±0.22) μA/cm^2^ and long spacers of 3.1 ± 0.31 μA/cm^2^. Glycine-coupled beads served as control and produced a slightly higher amount of current compared to the experiment without addition of riboflavin or magnetic beads (4.6 ± 0.47 μA/cm^2^). With short spacers, addition of riboflavin-coupled beads to the anodic chamber resulted in a 2.4-fold increase of the average current density compared to experiment without magnetic beads, whereas the glycine-coupled control only led to a minor increase (1.4-fold).

**FIGURE 3:**
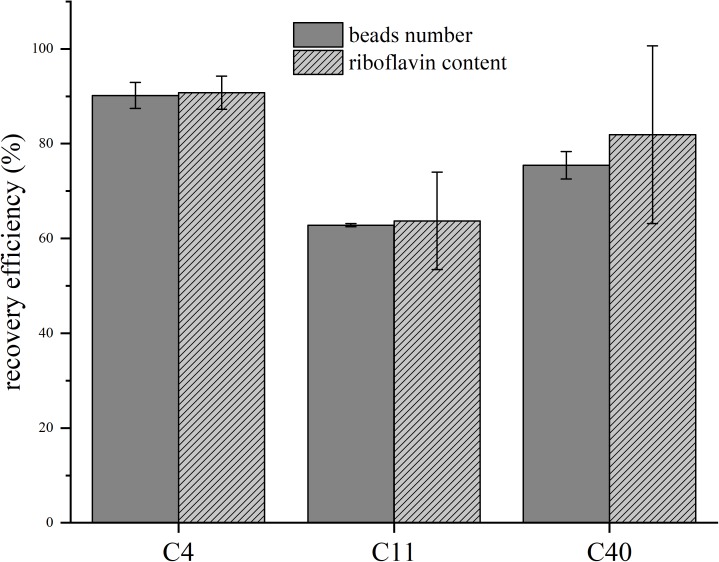
Recovery efficiencies of the beads and riboflavin content before and after batch experiments. C4/C11/C40 represent riboflavin-coupled beads with the respective chain lengths. Gray bars show the normalization to the beads number, striped bars to riboflavin content before the experiment and after the recovery process. Error bars represent the standard deviations from means of samples taken in independent triplicates.

We also analyzed the recovery rate and riboflavin concentration of all beads after the experiment and could measure for all setups very good recovery efficiencies of up to 90% (Figure 3). The stability of the riboflavin-coupled beads was analyzed via BES experiments in which the beads were recycled from the anode compartment and then reused in a fresh BES setup. The experiment was repeated three times and no significant change in the current density could be detected (data not shown).

### Riboflavin Coupling Does Not Lead to Robust Binding of Cells to Beads

From the previous results, we were interested, if riboflavin coupled to the beads binds to OMCs as cofactor. We compared MR-1 cells with a mutant, which is lacking all OMCs (ΔOMC) and added beads coupled to riboflavin and glycine, respectively. Cells were transferred from an oxic to an anoxic environment and beads were harvested after 15 h of incubation. In OMCs, the binding pocket for riboflavin is oxygen sensitive and closed under anoxic conditions (Edwards et al., 2015), so that a binding between riboflavin and OMCs should result in a co-elution of cells with the beads. However, microscopic analysis of all preparations exhibited no differences between wild type and ΔOMC cells (data not shown), which indicates that at least under the chosen conditions riboflavin does not seem to bind to the OMCs.

### Riboflavin Has a Pronounced Effect on Cell Distribution in BESs

**FIGURE 4:**
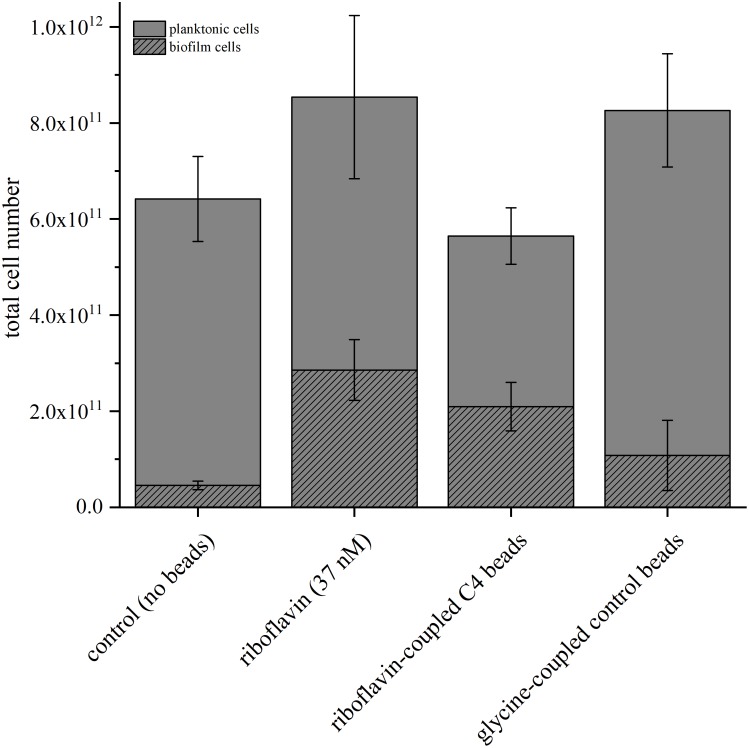
Comparison of the total cell number determined via qPCR in BES with the addition of riboflavin, glycine-coupled control beads and riboflavin-coupled beads with a linker length of 4 carbon atoms. The riboflavin-coupled beads were added so that the overall riboflavin concentration was equal to the experiment with free riboflavin (37 nM). The control experiment was conducted without addition of riboflavin or magnetic beads. Gray bars indicate planktonic cells; striped bars biofilm cells. Error bars represent the standard deviations from means of samples taken in independent triplicates.

We compared total cell numbers in the different experiments and quantified the number of planktonic vs. anode-attached cells to investigate the effect of riboflavin and magnetic beads on the cell distribution in the anode chamber in further detail. Therefore, qPCR analysis was conducted to quantify the number of planktonic and biofilm-attached cells in the BES containing soluble riboflavin, riboflavin coupled to C4-beads and glycine-coupled control beads (Figure 4). In BES without the addition of beads, the number of planktonic cells was comparable both with and without riboflavin. On the anode, the cell number increased significantly (*p* = 0.021) and 6.3-fold more cells could be detected compared to BES without the addition of riboflavin. Of note, this correlates well with the measured current increase. Adding riboflavin-coupled beads to the BES also influences the distribution of the cells. As seen with riboflavin as soluble compound, significantly more cells attached to the anode (*p* = 0.042), whereas with glycine-coupled beads cell adhesion was positively affected but with rather high standard deviations which rendered this effect to be not significant (*p* = 0.277). Again, the increase in anode attached cells correlates roughly with the stimulating effect of the beads on current production. Therefore, it can be assumed that there is a correlation of cells on the anode and current production and that this correlation is driven by riboflavin (either coupled to beads or as free compound), whereas the beads *per se* affect biofilm growth at least not as robustly.

### Following the Effect of Riboflavin-Coupled Beads in Flow Cells

**FIGURE 5:**
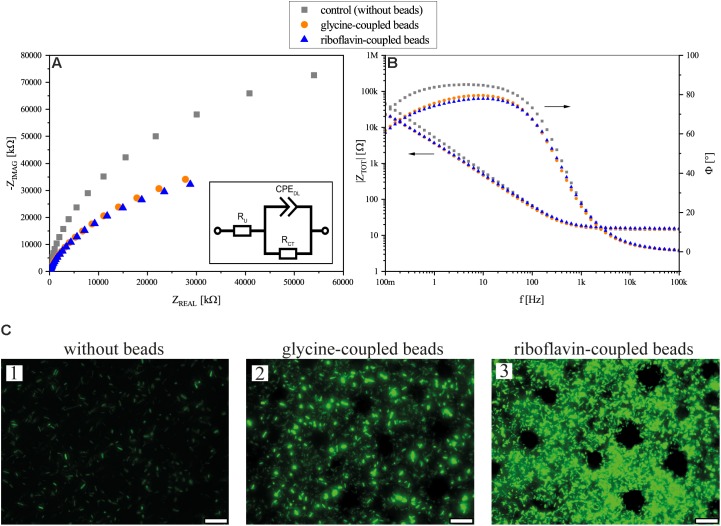
Flow cell experiment results. **(A)** Nyquist presentation of EIS measurements after 24 h of flow cell operation. **(B)** Bode plot and phase angle of the EIS measurements of flow cells without beads, with glycine-coupled beads, and with riboflavin-coupled beads. In both plots **(A,B)**, flow cells without beads are depicted in gray squares, flow cells with glycine-coupled control beads in orange circles, and flow cells with riboflavin-coupled beads in blue triangles. Points represent average values of independent triplicates; arrows indicate the corresponding axis. The inset in **(A)** shows an equivalent electrical circuit for the BES flow cell; R_CT_: charge transfer resistance; R_U_: solution resistance; CPE_DL_: double layer constant phase element. **(C)** Fluorescence microscopy images of *gfp*-expressing MR-1 on ITO glass anodes in experiments without beads (1), with glycine-coupled control beads (2) and with riboflavin-coupled beads (3) after 24 h of incubation. Circular areas without cells are due to the position of magnetic beads. The scale bar indicates 25 μm.

Continues flow BES experiments with a transparent ITO WE was performed to investigate the impedance of the biofilm and image it with fluorescence microscopy. The flow cells were incubated under potentiostatic conditions for 24 h after which EIS and fluorescence microscopy analyses were performed. In general, the data reveal a decrease of the total impedance of the BES with the addition of the beads as can be seen in both the Nyquist and Bode plots (Figure 5A,B). Both glycine- and riboflavin-coupled beads show almost identical trends in the Nyquist plot. The addition of glycine-coupled beads decreased the charge transfer resistance (i.e., R_CT_) from 164.7 kΩ (±19.1) in the control BES (without beads addition) to 74.8 kΩ (±11.6) and a relatively similar decrease in the R_CT_ was also observed after the addition of riboflavin-coupled beads [78.4 kΩ (±21.4)]. Fluorescence imaging was performed in order to illustrate the influence of the beads on the biofilm formation of MR-1 in the flow cell. Microscopic images taken in the flow cell (Figure 5C) show that the beads increase the attachment of the cells to the anode surface. As can be seen, even the addition of glycine-coupled beads leads to an increased GFP signal on the ITO WE. Furthermore, magnetic beads coupled with riboflavin gave an even higher fluorescence signal, indicating a promoting effect on the biofilm formation under anode respiring conditions.

## Discussion

In this study, an increased current density in MR-1-inoculated BESs was achieved by adding a recyclable electron shuttle composed of riboflavin-coupled magnetic beads. From an applied point of view the concentration of endogenous shuttles is usually rather low and exogenous addition of it is expensive and in some cases even environmentally toxic (Rinaldi et al., 2008). Additionally, permanent washout takes place in continuous cultivation systems. Hence, a shuttle that can be recycled as the here presented riboflavin-coupled magnetic beads would be advantageous.

In previous studies, shuttles were either added to the medium or produced endogenously by microorganisms. However, in all flow through experiments, the redox mediator has to be added continuously to avoid performance decrease due to washout. Using riboflavin-coupled magnetic beads, the beneficial effect of riboflavin can be recovered by magnetic separation and recycled with very good recovery efficiencies of up to 90%. This makes the process of adding bead-coupled riboflavin to BES much more sustainable, efficient and economical compared to the external addition or endogenous overproduction of the soluble compound.

The conducted experiments revealed a correlation between the increase of current and the number of cells on the anode for both free and bead-coupled riboflavin. Hence, the effect of riboflavin on current production is not necessarily due to a shuttling effect or its role as cofactor but could also be the result of a general biofilm promoting effect. These results are corroborated by a study from Bao et al. (2016) in which the authors could show that biofilm thickness of MR-1 on anodes can be increased by the addition of riboflavin. Interestingly, it does not seem to be necessary that the riboflavin is freely diffusible as the effect was detected also with riboflavin coupled to beads with a diameter of 25 μm. Still, the effect of riboflavin on biofilm and current production was higher when it was added in its free form. A possible factor for this might be that riboflavin coupled to the beads is not as accessible as the free compound. This limited accessibility might also be the reason, why we could not detect a binding of cells to the beads as a result of outer membrane cytochrome expression.

Impedance spectroscopy revealed that the addition of beads decreases the impedance by more than 50%. This decrease is mostly connected to the charge transfer resistance which is seen in the Nyquist plot as the beginning of a classical charge transfer semi-circle. The coupling of riboflavin (i.e., electroactive) or glycine (i.e., non-electroactive) did not show any significant difference in the charge transfer resistance. Since the beads are made out of iron oxide and covered by a presumably permeable agarose coating, it is possible that they are conductive themselves and can reduce the resistance of the anode by acting as an extension of the electrode, thereby extending the electroactive surface area and lowering the overall impedance. In this case, the covalently attached compound (riboflavin and glycine, respectively) does not seem to affect the electrochemical properties of the bioanode. However, the microscopic images indicate an increased biofilm production caused by the addition of beads to the system and regarding biofilm density, riboflavin-coupled beads clearly outperform glycine-coupled ones. This enhanced cell density is reflected in the qPCR data, where riboflavin-coupled beads also exhibits a boosting effect on the number of anode-attached cells.

## Conclusion

It is possible that the effect of the beads on a BES is dual: an improved conductivity leads to decreased resistance and by this more effective EET and current production, and (as stated above) riboflavin itself not only influences the electron transfer to the anode but also plays a role in biofilm production and/or formation.

## Author Contributions

TA, L-AP, DR, ME, JC, and MS carried out the experiments. KS-R wrote the manuscript with support from DR and JG. RU and DH supervised the electrochemistry part. JG supervised the project.

## Conflict of Interest Statement

The authors declare that the research was conducted in the absence of any commercial or financial relationships that could be construed as a potential conflict of interest.

## References

[B1] Babanova S. Matanovic I. Cornejo J. Bretschger O. Nealson K. Atanassov P. (2017). Outer membrane cytochromes/flavin interactions in *Shewanella* spp.-A molecular perspective. *Biointerphases* 12:021004. 10.1116/1.4984007 28565913 PMC5451299

[B2] Bao H. Zheng Z. Yang B. Liu D. Li F. Zhang X. (2016). In situ monitoring of *Shewanella oneidensis* MR-1 biofilm growth on gold electrodes by using a Pt microelectrode. *Bioelectrochemistry* 109 95–100. 10.1016/j.bioelechem.2016.01.008 26850925

[B3] Bartzatt R. Wol T. (2014). Detection and Assay of Vitamin B-2 (Riboflavin) in Alkaline Borate Buffer with UV/Visible Spectrophotometry. *Int. Sch. Res. Not.* 2014 1–7. 10.1155/2014/453085 27379273 PMC4897266

[B4] Beblawy S. Bursac T. Paquete C. Louro R. Clarke T. A. Gescher J. (2018). Extracellular reduction of solid electron acceptors by *Shewanella oneidensis*. *Mol. Microbiol.* 109 571–583. 10.1111/mmi.14067 29995975

[B5] Bond D. R. Lovley D. R. (2003). Electricity production by *Geobacter sulfurreducens* attached to electrodes. *Appl. Environ. Microbiol.* 69 1548–1555. 10.1128/AEM.69.3.1548-1555.2003 12620842 PMC150094

[B6] Brutinel E. D. Gralnick J. A. (2012). Shuttling happens: soluble flavin mediators of extracellular electron transfer in *Shewanella*. *Appl. Microbiol. Biotechnol.* 93 41–48. 10.1007/s00253-011-3653-0 22072194

[B7] Bucking C. Piepenbrock A. Kappler A. Gescher J. (2012). Outer-membrane cytochrome-independent reduction of extracellular electron acceptors in *Shewanella oneidensis*. *Microbiology* 158 2144–2157. 10.1099/mic.0.058404-0 22493303

[B8] Bursac T. Gralnick J. A. Gescher J. (2017). Acetoin production via unbalanced fermentation in *Shewanella oneidensis*. *Biotechnol. Bioeng.* 114 1283–1289. 10.1002/bit.26243 28059435

[B9] Chong G. W. Karbelkar A. A. El-Naggar M. Y. (2018). Nature’s conductors: what can microbial multi-heme cytochromes teach us about electron transport and biological energy conversion? *Curr. Opin. Chem. Biol.* 47 7–17. 10.1016/j.cbpa.2018.06.007 30015234

[B10] Costa N. L. Clarke T. A. Philipp L. A. Gescher J. Louro R. O. Paquete C. M. (2018). Electron transfer process in microbial electrochemical technologies: the role of cell-surface exposed conductive proteins. *Bioresour. Technol.* 255 308–317. 10.1016/j.biortech.2018.01.133 29444758

[B11] Dolch K. Wuske J. Gescher J. (2016). Genomic barcode-based analysis of exoelectrogens in wastewater biofilms grown on anode surfaces. *J. Microbiol. Biotechnol.* 26 511–520. 10.4014/jmb.1510.10102 26699756

[B12] Edwards M. J. White G. F. Norman M. Tome-Fernandez A. Ainsworth E. Shi L. (2015). Redox linked flavin sites in extracellular decaheme proteins involved in microbe-mineral electron transfer. *Sci. Rep.* 5:11677. 10.1038/srep11677 26126857 PMC4486940

[B13] Gorby Y. A. Yanina S. McLean J. S. Rosso K. M. Moyles D. Dohnalkova A. (2006). Electrically conductive bacterial nanowires produced by *Shewanella oneidensis* strain MR-1 and other microorganisms. *Proc. Natl. Acad. Sci. U.S.A.* 103 11358–11363. 10.1073/pnas.0604517103 16849424 PMC1544091

[B14] Javed M. M. Nisar M. A. Ahmad M. U. Yasmeen N. Zahoor S. (2018). Microbial fuel cells as an alternative energy source: current status. *Biotechnol. Genet. Eng. Rev.* 34 1–27. 10.1080/02648725.2018.1482108 29929427

[B15] Kotloski N. J. Gralnick J. A. (2013). Flavin electron shuttles dominate extracellular electron transfer by *Shewanella oneidensis*. *mBio* 4:e00553-12. 10.1128/mBio.00553-12 23322638 PMC3551548

[B16] Logan B. E. (2009). Exoelectrogenic bacteria that power microbial fuel cells. *Nat. Rev. Microbiol.* 7 375–381. 10.1038/nrmicro2113 19330018

[B17] Lovley D. R. Fraga J. L. Blunt-Harris E. L. Hayes L. A. Phillips E. J. P. Coates J. D. (1998). Humic substances as a mediator for microbially catalyzed metal reduction. *Acta Hydrochim. Hydrobiol.* 26 152–157. 10.1002/(SICI)1521-401X(199805)26:3<152::AID-AHEH152>3.0.CO;2-D

[B18] Lu M. Chan S. Babanova S. Bretschger O. (2017). Effect of oxygen on the per-cell extracellular electron transfer rate of *Shewanella oneidensis* MR-1 explored in bioelectrochemical systems. *Biotechnol. Bioeng.* 114 96–105. 10.1002/bit.26046 27399911 PMC5132103

[B19] Malvankar N. S. Vargas M. Nevin K. P. Franks A. E. Leang C. Kim B. C. (2011). Tunable metallic-like conductivity in microbial nanowire networks. *Nat. Nanotechnol.* 6 573–579. 10.1038/nnano.2011.119 21822253

[B20] Marsili E. Baron D. B. Shikhare I. D. Coursolle D. Gralnick J. A. Bond D. R. (2008). *Shewanella* secretes flavins that mediate extracellular electron transfer. *Proc. Natl. Acad. Sci. U.S.A.* 10.1073/pnas.0710525105 18316736 PMC2268775

[B21] Nealson K. H. Belz A. McKee B. (2002). Breathing metals as a way of life: geobiology in action. *Antonie Van Leeuwenhoek* 81 215–222. 10.1023/A:1020518818647 12448720

[B22] Nealson K. H. Rowe A. R. (2016). Electromicrobiology: realities, grand challenges, goals and predictions. *Microb. Biotechnol.* 9 595–600. 10.1111/1751-7915.12400 27506517 PMC4993177

[B23] Okamoto A. Kalathil S. Deng X. Hashimoto K. Nakamura R. Nealson K. H. (2015). Cell-secreted flavins bound to membrane cytochromes dictate electron transfer reactions to surfaces with diverse charge and pH. *Sci. Rep.* 4:5628. 10.1038/srep05628 25012073 PMC4092373

[B24] Okamoto A. Nakamura R. Nealson K. H. Hashimoto K. (2014). Bound flavin model suggests similar electron-transfer mechanisms in *Shewanella* and *Geobacter*. *ChemElectroChem* 1 1808–1812. 10.1002/celc.201402151

[B25] Polizzi N. F. Skourtis S. S. Beratan D. N. (2012). Physical constraints on charge transport through bacterial nanowires. *Faraday Discuss.* 155 43–62; discussion 103–114. 10.1039/C1FD00098E 22470966 PMC3392031

[B26] Reguera G. McCarthy K. D. Mehta T. Nicoll J. S. Tuominen M. T. Lovley D. R. (2005). Extracellular electron transfer via microbial nanowires. *Nature* 435 1098–1101. 10.1038/nature03661 15973408

[B27] Rinaldi A. Mecheri B. Garavaglia V. Licoccia S. Di Nardo P. Traversa E. (2008). Engineering materials and biology to boost performance of microbial fuel cells: a critical review. *Energy Environ. Sci.* 1:417 10.1039/b806498a

[B28] Shi L. Fredrickson J. K. Zachara J. M. (2014). Genomic analyses of bacterial porin-cytochrome gene clusters. *Front. Microbiol.* 5:657. 10.3389/fmicb.2014.00657 25505896 PMC4245776

[B29] Simonte F. Sturm G. Gescher J. Sturm-Richter K. (2017). *Extracellular Electron Transfer and Biosensors.* Berlin: Springer. 10.1007/10_2017_34 29071406

[B30] Stöckl M. Schlegel C. Sydow A. Holtmann D. Ulber R. Mangold K.-M. (2016). Membrane separated flow cell for parallelized electrochemical impedance spectroscopy and confocal laser scanning microscopy to characterize electro-active microorganisms. *Electrochim. Acta* 220 444–452. 10.1016/j.electacta.2016.10.057

[B31] Velasquez-Orta S. B. Head I. M. Curtis T. P. Scott K. Lloyd J. R. von Canstein H. (2010). The effect of flavin electron shuttles in microbial fuel cells current production. *Appl. Microbiol. Biotechnol.* 85 1373–1381. 10.1007/s00253-009-2172-8 19697021

[B32] White G. F. Edwards M. J. Gomez-Perez L. Richardson D. J. Butt J. N. Clarke T. A. (2016). Mechanisms of bacterial extracellular electron exchange. *Adv. Microb. Physiol.* 68 87–138. 10.1016/bs.ampbs.2016.02.002 27134022

[B33] Xu S. Jangir Y. El-Naggar M. Y. (2016). Disentangling the roles of free and cytochrome-bound flavins in extracellular electron transport from *Shewanella oneidensis* MR-1. *Electrochim. Acta* 198 49–55. 10.1016/j.electacta.2016.03.074

[B34] Zhai D. D. Li B. Sun J. Z. Sun D. Z. Si R. W. Yong Y. C. (2016). Enhanced power production from microbial fuel cells with high cell density culture. *Water Sci. Technol.* 73 2176–2181. 10.2166/wst.2016.059 27148719

